# Updates of the role of B-cells in ischemic stroke

**DOI:** 10.3389/fncel.2024.1340756

**Published:** 2024-03-14

**Authors:** Silin Wu, Sidra Tabassum, Cole T. Payne, Heng Hu, Aaron M. Gusdon, Huimahn A. Choi, Xuefang S. Ren

**Affiliations:** Division of Neurocritical Care, Department of Neurosurgery, McGovern School of Medicine, University of Texas Health Science Center, Houston, TX, United States

**Keywords:** ischemic stroke, inflammatory response, B-cell, T-cell, immune cells, immunotherapy

## Abstract

Ischemic stroke is a major disease causing death and disability in the elderly and is one of the major diseases that seriously threaten human health and cause a great economic burden. In the early stage of ischemic stroke, neuronal structure is destroyed, resulting in death or damage, and the release of a variety of damage-associated pattern molecules induces an increase in neuroglial activation, peripheral immune response, and secretion of inflammatory mediators, which further exacerbates the damage to the blood–brain barrier, exacerbates cerebral edema, and microcirculatory impairment, triggering secondary brain injuries. After the acute phase of stroke, various immune cells initiate a protective effect, which is released step by step and contributes to the repair of neuronal cells through phenotypic changes. In addition, ischemic stroke induces Central Nervous System (CNS) immunosuppression, and the interaction between the two influences the outcome of stroke. Therefore, modulating the immune response of the CNS to reduce the inflammatory response and immune damage during stroke is important for the protection of brain function and long-term recovery after stroke, and modulating the immune function of the CNS is expected to be a novel therapeutic strategy. However, there are fewer studies on B-cells in brain function protection, which may play a dual role in the stroke process, and the understanding of this cell is still incomplete. We review the existing studies on the mechanisms of the role of B-cells, inflammatory response, and immune response in the development of ischemic stroke and provide a reference for the development of adjuvant therapeutic drugs for ischemic stroke targeting inflammatory injury.

## 1 Introduction

Stroke is a major global health concern, ranking as the second leading cause of death and a major cause of disability worldwide (Safiri et al., 2021; Collaborators, 2023). It is divided into ischemic stroke and hemorrhagic stroke, of which ischemic stroke accounts for the majority, and ischemic stroke accounts for about 80% of all cerebrovascular diseases, which is the main cause of serious neurological damage and harm, and it has a very high rate of disability, death, and recurrence (Feigin et al., 2014; Aguiar de Sousa et al., 2019; Collaborators, 2023). Its incidence varies considerably across different regions, with higher rates often observed in low- and middle-income countries. The epidemiology of stroke is influenced by a complex interplay of genetic, environmental, and lifestyle factors. Common risk factors include hypertension, smoking, diabetes, obesity, and heart diseases (Collaborators, 2023).

Currently, the fundamental treatment for ischemic stroke is tissue-type plasminogen activator (tPA) thrombolysis or combined with endovascular thrombectomy aimed at restoring reperfusion and improving recanalization rates in a timely and effective manner, but the narrow therapeutic window, risk of complications, and limited therapeutic efficacy limit access to treatment to only a small number of patients (National Institute of Neurological, D., and Stroke rt, P.A.S.S.G, 1995; Liu et al., 2023; She et al., 2023). Advancements in the treatment and research of stroke have been significant over the past decades. The introduction of thrombolytic therapy, specifically tPA, marked a turning point in acute ischemic stroke management. This treatment, when administered within a specific time window after stroke onset, can effectively dissolve the blood clot causing the stroke, thereby restoring blood flow to the affected brain region (Liu et al., 2023). However, its utility is limited by the narrow therapeutic time window and contraindications in certain patients. Mechanical thrombectomy, another breakthrough, has transformed care for certain types of strokes. This procedure involves the physical removal of a clot from a blocked blood vessel in the brain and has been shown to significantly improve outcomes in patients with large vessel occlusions. It also has a longer therapeutic window compared to tPA (Guzauskas et al., 2017).

Research in stroke has expanded into exploring the role of neuroprotection, rehabilitation techniques, and the potential of stem cell therapy. Neuroprotective agents aim to protect the brain tissue from damage following a stroke, although successful translation from laboratory to clinic is still a challenge (Paul and Candelario-Jalil, 2021). Rehabilitation, including physical, occupational, and speech therapy, is essential for improving functional outcomes after a stroke (Feigin et al., 2014). Ren et al. demonstrated in tMCAO mice that replacing 20% of the blood of stroke mice (8–12 months) with blood from young mice (3–6 months) 6.5–7 h after stroke significantly attenuates the latter’s stroke symptoms, reduces the infarcted volume of the brain, and improves their neurological deficits (Ren et al., 2020). This is the first study to show that blood replacement therapy improves stroke outcome in mice, providing new insights into the mechanisms of stroke injury and may lead to new breakthroughs in stroke treatment in the clinic (Ren et al., 2020). Recent research has also delved into the genetic underpinnings of stroke, aiming to identify genetic markers that could predict stroke risk or influence treatment response (Boehme et al., 2017). There is ongoing investigation into the use of artificial intelligence and machine learning in stroke diagnosis and prognosis, which promises to enhance the precision and efficiency of stroke care (van Asch et al., 2010). Despite these advancements, stroke remains a significant challenge due to its sudden onset, the potential for severe disability, and the aging global population, which is likely to increase the burden of this disease. Continuous research and improvements in public health strategies are essential to reduce its incidence and impact. In recent years, many studies have confirmed that the inflammatory response and the central nervous system intrinsic and peripheral immune responses play a key role in the regulation of ischemic stroke, which triggers the activation of microvascular endothelial cells and the leakage of the blood–brain barrier after ischemia and is accompanied by the diffusion of various inflammatory mediators along the cerebrospinal fluid or interstitial fluid, which leads to the emergence of activated glial cells with infiltrating B-cells, T-cells, and other peripheral immune cells in infarcted foci and the peripheral tissue (Malone et al., 2019; Shi et al., 2019). This leads to the emergence of activated glial cells in the infarct and peripheral tissues and the infiltration of various peripheral immune cells, including B-cells and T-cells, resulting in changes in the brain microenvironment and further triggering the inflammatory response environment in the brain (Mracsko et al., 2014; Liesz and Kleinschnitz, 2016). Therefore, the importance of central and peripheral immune responses to stroke has become a hotspot of widespread concern and is expected to become a new therapeutic target, immunization, but these studies are still in the experimental stage and still face many problems and difficulties in the actual clinical treatment (Mracsko et al., 2014; Gadani et al., 2015). The aim of this paper is to summarize the current research progress on the mechanism and treatment of B-lymphocytes and stroke and to provide a reference for seeking new clinical therapeutic strategies.

## 2 Overview of the role of B-cells in brain function and disease

The brain’s immune system is a complex and specialized network of multiple immune cells and components responsible for protecting the brain from injury and disease (Mracsko and Veltkamp, 2014). These major immune cells and components include microglia, astrocytes, phagocytes in the brain, peripheral immune cells, and cytokines and chemokines. Under homeostatic conditions of the internal environment, many immune cells are found mainly in the meninges and choroid plexus; however, Posel et al. also found some lymphocytes in the brain parenchyma, e.g., in the hippocampus, where partial lymphocyte reaggregation was found (Posel et al., 2016). In the normal brain and in the uninjured adult brain, microglia play an important role in maintaining brain homeostasis, synaptic connectivity, and remodeling of brain function as well as in monitoring damaged or dead neuronal cells and foreign bodies throughout the brain (Emsley and Tyrrell, 2002; Prinz and Priller, 2014). Microglia are the main specific immune cells of the brain, the resident immune cells of the central nervous system, and the first direct responders to initiate cerebral protection after brain injury. Microglia fulfill a surveillance function in the healthy brain and are activated in pathological conditions such as infection or injury to participate in the inflammatory response (Reemst et al., 2016; Vainchtein et al., 2018). In the brain, the neuroinflammatory response is characterized by microglia activation, inflammatory cell aggregation, leukocyte infiltration, and the subsequent release and further recruitment of cytokines, chemokines, and a variety of neurotoxic molecules, which in turn trigger protective mechanisms secondary to brain injury (Emsley and Tyrrell, 2002; Yang et al., 2016). Astrocytes are the predominant neuro-skeletal support cells in the brain, and although they are primarily neural support cells, they are extremely abundant. Astrocytes also play a role in immune responses, such as regulating the integrity of the blood–brain barrier, as well as the release of cytokines, chemokines, and axon guidance, neuronal survival and function, maintenance of the blood–brain barrier, regulation of cerebral blood flow, and secretion of neuroprotective factors during inflammation and repair (Wu et al., 2015; Scimemi, 2018; Vainchtein et al., 2018). After an ischemic stroke, astrocytes proliferate in large numbers, but their exact function remains unclear. After brain injury, activated astrocytes can promote innate immune responses to some extent, exhibiting a double-edged effect that is both beneficial and detrimental to brain function (Blackburn et al., 2009; Scimemi, 2018). These activated reactive astrocytes proliferate after brain injury and sustain the release of a variety of pro- and anti-inflammatory cytokines, which in turn can further inversely regulate microglial activation or reactive processes (Sukumari-Ramesh et al., 2012; Scimemi, 2018). Intracerebral macrophages are present in brain tissue and help to remove dead and damaged cells (Prinz and Priller, 2014).

Peripheral immune cells are usually located outside the brain, but in some cases, such as brain injury or inflammation, they can cross the blood–brain barrier into the brain (Kleinschnitz et al., 2013). These cells include T-cells, B-cells, and peripheral macrophages. B-cells play multiple important roles in the adaptive immune system, not only playing a dominant role in antibody-mediated immune responses but also playing a key function in immune regulation and the formation of immune memory. When B-cells recognize and bind to specific antigens, they differentiate into plasma cells. Plasma cells are specialized antibody-producing cells that make antibodies against specific antigens in large quantities and rapidly capture specific antigens via B-cell receptors (BCRs) on their surface, internalize them and process them, and then display antigen fragments on their surface via MHC II molecules (Barroso et al., 2015; Huszthy et al., 2019; Brown et al., 2020). In addition, B-cells act synergistically with T-cells, with antigen-displaying B-cells interacting with specific helper cells (T helper cells), a process that is essential for B-cell activation and differentiation (Huszthy et al., 2019). B-cells also produce and release cytokines, molecules that play an important role in regulating immune responses, inflammatory reactions, and communication with other immune cells (Matejuk et al., 2021). Through these cytokines, B-cells can influence the function of other immune cells, such as by promoting or inhibiting T-cell activity, which in turn regulates the intensity and type of immune response. Cytokines and chemokines are signaling molecules produced by immune cells and glial cells that play a key role in regulating immune responses and inflammation (Emsley and Tyrrell, 2002; Chang et al., 2018; Matejuk et al., 2021). The brain’s immune system differs from that of the rest of the body in that it must protect the brain from infection while maintaining its complex functional and structural integrity. As a result, the regulation and function of the brain’s immune system is highly specialized and refined, affecting the course of a stroke in a variety of ways.

The immune response to an ischemic stroke is a complex process involving multiple immune cells and mechanisms. B-lymphocytes respond rapidly after ischemic injury to the brain, but their actions tend to be bipartite, either harmful or beneficial, and are particularly prominent in patients with comorbid hypertension, coronary artery disease, diabetes mellitus, and advanced age (Doyle et al., 2015; Doyle and Buckwalter, 2017; Ortega et al., 2020). Schuhmann et al. found that in the early stages of acute ischemic stroke, B-cells have a very limited effect on stroke lesion volume and functional recovery (Schuhmann et al., 2017). However, on the other hand, a number of research groups have reported that B-lymphocytes, especially regulatory B-cells (Bregs), exhibit beneficial effects in ischemic stroke, such as neuroprotection, neuro-recovery, neurotrophic, and cleansing of damaged cellular components, among others (Ren et al., 2011a; Offner and Hurn, 2012). A study by McCulloch et al. reported that stroke leads to a decrease in B-cells and immunoglobulin IgM in patients, increasing the risk of infection, which can be mitigated using β-adrenergic receptor antagonists (McCulloch et al., 2017). Ortega et al. found that B-cells contribute to stroke recovery; they migrate to different regions of the brain and promote functional recovery; and that the lack of B-cells is associated with delays in the recovery process, showing an important role for B-cells in neuro-recovery and neuroprotection after stroke (Ortega et al., 2020). Korf et al. explored the role of B-cells with high expression of CD11b in the immune response after stroke and found that these cells, which were increased in the brains of aged and post-stroke mice, were able to modulate the phenotype and increase the phagocytosis of microglial cells, which in turn had an impact on neuroinflammation after stroke, suggesting that B-cells have a distinctive role for specific subpopulations in both the acute and chronic phases of the immune response after stroke (Korf et al., 2022). Ren et al. found that the use of blood replacement helped to improve stroke in mice and found that the total number of leukocytes and neutrophils in the blood of stroke mice was significantly reduced during and 1 h after BR treatment, and that BR treatment significantly reduced plasma levels of the pro-inflammatory cytokines IL-1β, IL-6, and TNF-α, as well as of the chemokine CXCL1, in stroke mice (Ren et al., 2020).

Although the specific role and function of activated microglia have not been fully clarified, some studies have shown that activated microglia can trigger and release cytokines, chemokines, prostaglandins, proteases, ferrous iron, glutamate, and reactive oxygen species after ischemic stroke (Emsley and Tyrrell, 2002; Prinz and Priller, 2014). The release of these inflammatory mediators and activation of the intrinsic immune response further led to increased permeability of the blood–brain barrier, which allows peripheral immune cells such as B-cells, T-cells, and inflammatory factors to enter the brain tissue. Astrocytes are the most important neuroskeletal supporting cells in the brain, and although they are mainly neural supporting cells, they are extremely abundant. After an ischemic stroke, astrocytes proliferate massively, but their exact function is still unclear. They may induce B-cells and T-cells to enter the brain by continuously releasing a variety of pro-inflammatory and anti-inflammatory cytokines (Sukumari-Ramesh et al., 2012; Scimemi, 2018). Increased apoptosis and decreased or dysfunctional immune cells, such as monocytes, T-lymphocytes, B-lymphocytes, and natural killer (NK) cells, can be observed in the early stages of stroke, and these changes often indicate a poor prognosis (Offner et al., 2006; Liesz et al., 2015). After the onset of an ischemic stroke, B-cells migrate to the brain as part of the immune response. After stroke, the infiltration of T-lymphocytes at the site of brain injury varies depending on their proinflammatory or anti-inflammatory function (Kim et al., 2021). Studies have shown that activated T-lymphocytes play a detrimental role in the early stage of ischemic cerebral infarction, while Tregs, as endogenous regulators with potential neuroprotective effects, play a protective role in blood–brain barrier permeability, leukocyte infiltration, tissue edema, and brain injury and play an important role in prognosis recovery (Kim et al., 2021).

Intrinsic B-cell-based immune responses play a dual role in ischemic stroke; inflammation in the acute phase may be detrimental to stroke recovery, but long-term inflammation is also necessary for tissue repair and remodeling (Kim et al., 2014; Engler-Chiurazzi et al., 2020; Malone et al., 2023). Proper understanding and regulation of B-lymphocyte function is essential to improve therapeutic strategies in ischemic stroke, and the balance and regulation of these responses are critical to the clinical prognosis of ischemic stroke.

## 3 The role of B-cells at the acute phase after stroke

After the occurrence of an acute ischemic stroke, the brain triggers a series of inflammatory reactions and immune response mechanisms (Frijns and Kappelle, 2002; Cervera et al., 2004; Engler-Chiurazzi et al., 2020). This process is often due to the sudden narrowing or obstruction of the responsible vessel, resulting in insufficient local blood supply or cessation of blood flow, leading to altered shear stress in the microvascular wall, insufficient local oxygenation, and the generation of large amounts of reactive oxygen species (ROS), which stimulate the vascular endothelial cells and circulating leukocytes to further activate the platelet and coagulation cascade reaction, rapidly activate the coagulation system, and cause microvascular obstruction (Boehme et al., 2016; Fam et al., 2017; Wium-Andersen et al., 2017). In the early phase of ischemic stroke, the immune activation process includes overall immunosuppression of the spleen, thymus, lymph nodes, and circulation. In the middle cerebral artery occlusion (tMACO) mouse model, it can be observed that extensive infarct foci induce a massive decrease in the number of peripheral blood leukocytes, splenic, lymph node, and thymic lymphocyte counts and predispose to poststroke infections, while smaller infarct foci do not have a significant effect on the number of peripheral blood cells and lymphocyte counts (Offner et al., 2006; Simard et al., 2007; Seifert and Offner, 2018; Kadry et al., 2020). Greater ischemic injury and higher mortality were observed in mice lacking mature B-lymphocytes, and transplantation of B-lymphocytes improved the prognosis of mice (Doyle and Buckwalter, 2017; Kim and Cho, 2021; Endres et al., 2022).

### 3.1 Activation and recruitment of B-cells

During the acute phase after stroke, dead or damaged neurons continue to release a variety of damage-associated molecules that further activate microglia and astrocytes. These activated glial cells release inflammatory mediators, such as cytokines and chemokines, which contribute to the inflammatory response (Vandenbark et al., 2021). Microglia are the first direct responders to initiate cerebral protection after brain injury and are activated in pathological conditions such as infection or injury to participate in the inflammatory response (Reemst et al., 2016; Vainchtein et al., 2018). After an ischemic stroke, astrocytes proliferate in large numbers, but their exact function remains unclear. Hernandez et al. found that microglial cells responded rapidly within 4 h after stroke, and in the hyperacute phase, astrocytes exhibited features marked by stress-responsive genes and transcription factors (e.g., Fos and Jun), which are involved in pro-inflammatory pathways such as TNF-α (Hernandez et al., 2023). After brain injury, activated astrocytes can promote the innate immune response to some extent, exhibiting a double-edged effect that is both beneficial and detrimental to brain function (Blackburn et al., 2009; Scimemi, 2018; Prescott et al., 2023).

These changes in the blood, vasculature, blood flow, and perimicrovascular space are superimposed on a series of related events, such as inadequate adenosine triphosphate (ATP) supply, mitochondrial dysfunction, bioenergetic failure, ionic homeostasis disorders, oxidative/nitrooxidative stress, acidosis, excitatory toxin release, and initiation of an inflammatory response in the brain parenchyma and brain cells, leading to neuronal death or damage and the formation of a focal ischemic core and peripheral ischemic semi-dark zones. At this point, dead and/or damaged neurons begin to release injury-associated pattern molecules, which are activated along with pattern recognition receptors on the surface of immune cells and produce pro-inflammatory factors such as interleukin-1β (IL-1β) and tumor necrosis factor-α (TNF-α), creating an early inflammatory milieu in the localized ischemic regions of the brain (Dardalhon et al., 2008; Liu et al., 2020). The inflammatory response further exacerbates vasoconstriction and platelet aggregation as well as disruption of vascular endothelial tight junctions, which leads to an increase in blood–brain barrier permeability, allowing peripheral immune cells (e.g., T-cells, B-cells, and macrophages) to cross the blood–brain barrier into the brain tissue (Dardalhon et al., 2008; Joshi et al., 2019; Endres et al., 2022). Several hours after cerebral ischemia, peripheral immune cells more readily enter the brain and participate in the local immune response as blood–brain barrier permeability increases. These cells can further amplify the inflammatory response, triggering leukocyte infiltration, extracellular proteolysis, and vascular activation, and may also be involved in the clearance and repair of damaged tissue (Dardalhon et al., 2008; Knowland et al., 2014; Joshi et al., 2019). The disruption of blood brain barrier (BBB) structure and increased permeability, as well as the release of various danger/damage-associated molecular patterns (DAMPs), induces peripheral immune cells to recruit and infiltrate into the lesion and its surroundings and to enter the systemic circulation through the damaged BBB or CSF drainage into the lymphatic vessels, which are extensively involved in the inflammatory and immune response process in ischemic stroke (Liesz et al., 2015; Bower and Hogan, 2018; Thadathil et al., 2021). Ultimately, the adaptive immune system is triggered and activated by damage-associated molecular patterns (DAMPs) that play a key role by binding to pattern recognition receptors (PRRs) such as Toll-like receptors (TLRs), scavenger receptors (e.g., CD36 and CD14), and other receptors on the surface of antigen-presenting cells such as dendritic cells and macrophages. This process involves the activation and differentiation of immune cells, leading to the generation of specific immune responses, which are essential for recognizing, responding to, and clearing pathogens or damaged tissue (Liesz et al., 2015).

### 3.2 Extensive activation of inflammatory factors

It is worth emphasizing that in the early acute phase after stroke, the inflammatory response is driven primarily by cells of the intrinsic immune system, such as macrophages, neutrophils, and microglia. These cells act by releasing inflammatory mediators, such as cytokines and chemokines, and by producing reactive oxygen species (ROS). Also, at this stage, lymphocytes, including B and T-cells, are involved in the response, and they exacerbate the inflammatory response by releasing cytokines and producing ROS, a process that does not depend on the specific recognition of a particular antigen (Kim et al., 2014). Stroke-induced peripheral inflammatory response plays an important role in determining neurological prognosis, and Katie et al. found that the peripheral inflammatory response peaks 4 h after stroke, earlier than the 24 h peak of cerebral inflammation, and is mainly characterized by an increase in the chemokine CXCL-1 and the pro-inflammatory cytokines interleukin-6 and IFN-γ (Chapman et al., 2009). Garaschuk and Verkhratsky found that in permanent middle cerebral artery occlusion (pMCAO) animal experiments, fragments of microglia expressing CD11b^+^ were detected in the infarcted area after 12 h of ischemia, followed by 2–3 days of activated microglia peaking at the site of ischemia and persisting for several weeks (Garaschuk and Verkhratsky, 2019).

**Figure 1:**
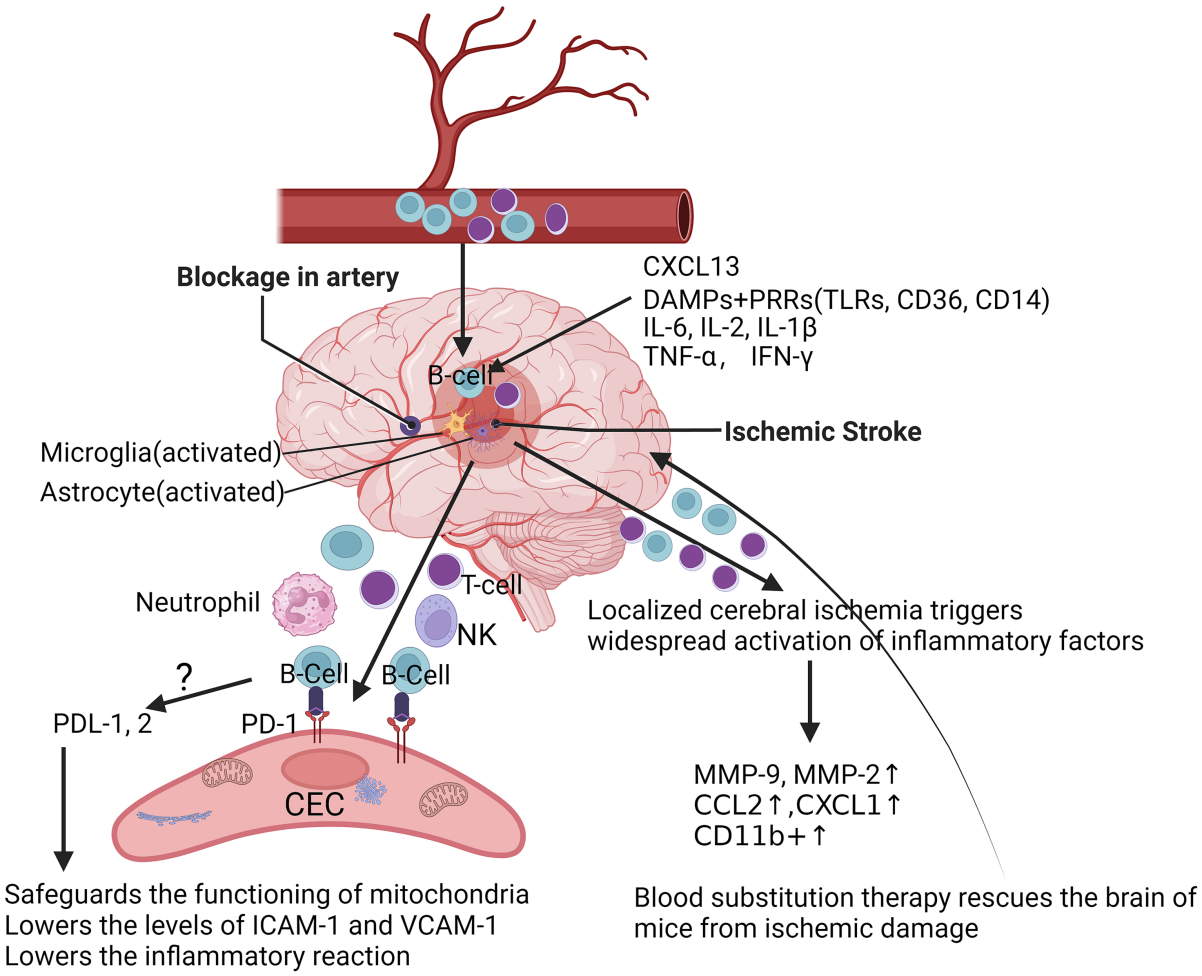
At the acute phase after ischemic stroke. After acute ischemic stroke occurs, resulting in inadequate local blood supply or cessation of blood flow, dead or damaged neurons continue to release a variety of damage-associated molecules, which further activate microglia and astrocytes, and DAMPs are triggered and inhibited by binding to PRRs, such as TLRs, scavenger receptors and lymphatic vessels, triggering and activating the adaptive immune system, followed by an increase in the chemokine CXCL-1 and the pro-inflammatory cytokines IL-6 and IFN-γ, as well as CD11b^+^, which further triggers a widespread activation of the peripheral immune system after stroke, with an increase in the production of IL-6, IL-2, TNF-α, IFN-γ, and CCL2 in splenic and circulating immune cells. After localized cerebral ischemia, the levels of MMP-2 and MMP-9 were significantly elevated in the rat brain, which markedly attenuated brain injury in stroke mice during and 1 h after Blood substitution treatment. Mice lacking PD-1 had more severe infarcts and more severe inflammatory responses after tMCAO. DAMPs, danger/damage-associated molecular patterns; TLRs, toll-like receptors; PRRs, pattern-recognition receptors; CXCL-1, C-X-C motif chemokine ligand 1; IL-6, interleukin-6; IL-2, interleukin-2; TNF-α, tumor necrosis factor; IFN-γ, interferon-gamma receptor; C-C motif chemokine ligand 2, CCL2; MMP-2, matrix metalloproteinase-2; MMP-9, matrix metalloproteinase-9; PD-1, programmed cell death protein 1; PD-L1/2, programmed cell death ligand 1/2; tMCAO, transient middle cerebral artery occlusion.

Localized cerebral ischemia (MCAO) triggers dynamic and widespread activation of inflammatory factors (including cytokines, chemokines, and chemokine receptors) in the peripheral immune system after stroke, with increased production of IL-6, IL-2, TNF-α, IFN-γ, and CCL2 in splenic and circulatory immune cells (Offner et al., 2006). Over time, immune cells may undergo phenotypic shifts. For example, microglia and macrophages shift from a phenotype that promotes inflammatory responses to one that promotes tissue repair (Figure 1). With increasing permeability of the blood–brain barrier, this would prompt vascular endothelial cells to detach from the basement membrane, ultimately leading to the free and unimpeded entry of water and serum into the brain, triggering secondary brain injury. Different cell types often play different roles in the injury and repair process after ischemic stroke to more effectively regulate post-stroke inflammation (Bramlett and Dietrich, 2004; Kim et al., 2014; Petrovic-Djergovic et al., 2016). Following localized cerebral ischemia, a significant increase in the activity of matrix metalloproteinases (MMPs) in the rat brain occurs in two temporal phases: first, an induced increase in MMP-9 from 4 h to 4 days, followed by a large increase in MMP-2 appearing on day 4, which may be related to MMP-2 in reactive microglial cells or macrophages (Planas et al., 2001). Ning et al. found that in patients with acute ischemic stroke, plasma levels of MMP9 were significantly elevated in patients treated with tPA in the early post-stroke period and correlated with poor prognosis (Ning et al., 2006). Ren et al. significantly improved stroke in mice using blood replacement and found that the total number of leukocytes and neutrophils in the blood of stroke mice was significantly reduced during and 1 h after BR treatment, and that BR treatment significantly reduced plasma levels of the pro-inflammatory cytokines IL-1β, IL-6, and TNF-α, as well as the chemokines CXCL1 and MMP-9 in stroke mice (Ren et al., 2020) (Figure 1). A recent clinical study has shown that poor stroke outcome modules are enriched with down-regulated B-cell-specific genes in stroke patients identified by whole-transcriptome analyses (Amini et al., 2023).

### 3.3 Pathways of B-cells into the brain

Though the pathways by which B-lymphocytes protect the brain after ischemic stroke remain unclear, one way is that after stroke, B-cells enter the brain through bilateral blood vessels and produce pro- or anti-inflammatory cytokines, depending on their subtype, which can affect T-cell-mediated inflammation and neuronal survival (Ortega et al., 2020; Zhang et al., 2021). Rayasam et al. demonstrated that IL-21-producing CXCR5^+^ CD4^+^ ICOS-1^+^ T follicular helper (TFH) cells are recruited in the brain to promote stroke injury after ischemic stroke. IL-21 activated the JAK/STAT pathway and induced apoptosis in primary ischemic neurons in mice under *in vitro* conditions, and inhibition of CXCL13 limited the migration and impact of these cells in the ischemic brain, thereby protecting mice from stroke injury (Rayasam et al., 2022). Nancy et al. found that repetitive hypoxic preconditioning (RHP) was able to alter the state of B-cells prior to stroke, creating an anti-inflammatory environment that could help to minimize brain damage after stroke (Monson et al., 2014). Altered BBB permeability and functional abnormalities may lead to increased lymphocyte density in brain tissue. Konstantin et al. found that in patients with schizophrenia, high inflammation levels were associated with blood–brain barrier dysfunction and increased immune cell migration (Schlaaff et al., 2020). The CNS is often regarded as an immune-privileged region, and several studies are now available that reveal a variety of known alterations in lymphocyte infiltration of the CNS, with a variety of specific molecules being involved in the process of lymphocyte expansion, including the P-selectin glycoprotein ligand (PSGL) and the vascular cell adhesion molecule (VCAM), as well as the intercellular adhesion molecule ICAM-1/2 (Carson et al., 2006; Keach et al., 2016) (Figure 1). Cai et al., reported that the permeability of the BBB was significantly altered, and this change was mainly due to the high expression of ICAM and the massive infiltration of cells of the peripheral immune system, such as macrophages (Cai et al., 2020). In addition, B-cells were activated and able to enter the parenchymal cells of the brain in cases of CNS infection (Keach et al., 2016). Our data suggest programmed cell death protein-1 (PD-1) is upregulated in CECs following ischemia (data not shown). PD-L1 and PD-L2 are significantly increased in B-cells after stroke (Ren et al., 2011b). Possibly, B-cells inhibit the reactions of CECs to ischemia through the PDL-1/2-PD-1 pathway (Figure 1), which is a currently uninvestigated mechanism.

In addition to the vascular route, B-cells may also enter the CNS through the blood-cerebrospinal fluid barrier (BCSFB) of the choroid plexus, a process that involves two steps of crossing the capillary and epithelial barriers. Disruption of the BBB is not only one of the critical pathways leading to hemorrhagic transformation after ischemia, but it further exacerbates the extent of CNS damage. When the integrity of the BBB is compromised, it allows substances in the blood, such as cells, proteins, and water, to penetrate brain tissue, which can lead to localized inflammation, tissue swelling, and other pathological changes. This disruption may also promote the accumulation of neurotoxic substances, further damaging nerve cells and exacerbating ischemic brain damage (Hong et al., 2021; Matejuk et al., 2021). Jürgen et al. found enhanced B-cell responses to specific chemokines such as CXCL-12/CXCL-13, especially in already activated memory B-cells. These cells exhibited enhanced chemotactic activity during migration through paracellular and transcellular pathways. The study also found that migration of such memory B-cells was reduced in multiple sclerosis (MS) patients treated with natalizumab, suggesting an emphasis on the role of BCSFB as an important channel for activated B-cells to enter the cerebrospinal fluid (Haas et al., 2020). The important role of B-cells in diseases of the central nervous system (CNS), particularly in multiple sclerosis, emphasizes the ways in which B-cells contribute to neuropathology by secreting antibodies and neurotoxic molecules as well as presenting antigens (Rodriguez-Mogeda et al., 2022).

Although there are few B-cells in the choroid plexus in healthy states, in neuroinflammatory conditions, such as multiple sclerosis, the number of B-cells, especially plasma cells, increases in the choroid plexus (Simard et al., 2007; Jain and Yong, 2022). In addition, Llovera et al. found that the choroid plexus (ChP) is a key pathway for T-cell invasion into the brain after stroke, especially after cerebral ischemia, with specific accumulation of T-cells in the peri-stroke cortical region and the ipsilateral ChP, and found that the CCR2 ligand gradient may be the driving force for this migration and depicted the neuroanatomical pathways of migration within the brain (Llovera et al., 2017). Currently, no studies have been reported on the mechanism of B-cell entry into the brain through the choroid plexus in the setting of stroke, and this area requires further investigation. In addition, it has been shown that transplantation of IL-10-secreting B-cells into wild-type mice with normal B-cell function results in a reduction of infarct size and improvement of neurological deficits before, after 4 h, or after 24 h of the establishment of the mouse MCAO model (Bodhankar et al., 2014, 2015). Ren et al. found that in a mouse model of stroke (MCAO), μMT^(−/−)^ mice lacking B-cells displayed larger infarct volumes, higher mortality, more severe dysfunction, and more activated T-cells, macrophages, microglial cells, and neutrophils compared with wild-type (WT). However, transfer of IL-10-secreting WT B-cells into μMT^(−/−)^ mice completely blocked these changes, whereas transfer of B-cells from IL-10^(−/−)^ mice was ineffective. These findings strongly support a role for IL-10-secreting B-cells in limiting infarct volume after stroke, reducing mortality, inhibiting inflammatory cell recruitment, and ameliorating functional neurologic injury, suggesting that regulatory B-cell enhancement may serve as a novel therapy for this severe neuropathic state (Ren et al., 2011a). Schuhmann et al. found that drug depletion of B-cells did not alter infarct volume or functional recovery on day 1 after stroke; similarly, lack of circulating B-cells did not affect recovery after stroke in the JHD^−/−^ and Rag1^−/−^ mouse models, suggesting that B-cells are not a major pathophysiologic factor and have a limited effect on lesion volume and functional recovery (Schuhmann et al., 2017).

These studies further highlight the diversity and complexity of B-cell responses after stroke. At different stages and settings of stroke, depending on their subtype, activation status, and interactions with other immune cells (Engler-Chiurazzi et al., 2020). Therefore, a deeper understanding of the specific role of B-cells in stroke is crucial for the development of targeted therapeutic strategies.

## 4 The role of B-cells at the chronic phase after stroke

Ischemic stroke is a multistage immune response process, and B-lymphocytes, as important immune cells, play a key role in the long-term course of ischemic stroke (Liesz and Kleinschnitz, 2016). It has been shown that B-cells are retained in the brain for up to 10 weeks in mouse models of stroke, whereas in human ischemic stroke patients, the increase in B-cells in the periphery lasts up to 12 weeks (Li et al., 2021; Weitbrecht et al., 2021). In the mouse model, B-cell transfer reduced infarct volume after stroke, independent of changes in the immune population of recipient mice. There is evidence that B-cells may be involved in the plasticity and regeneration of motor areas of the brain (Li et al., 2021). In addition, mice with bilateral removal of B-cells in the acute phase (4 days after stroke) showed increased migration of B-cells to brain regions associated with motor function (Li et al., 2021).

**Figure 2:**
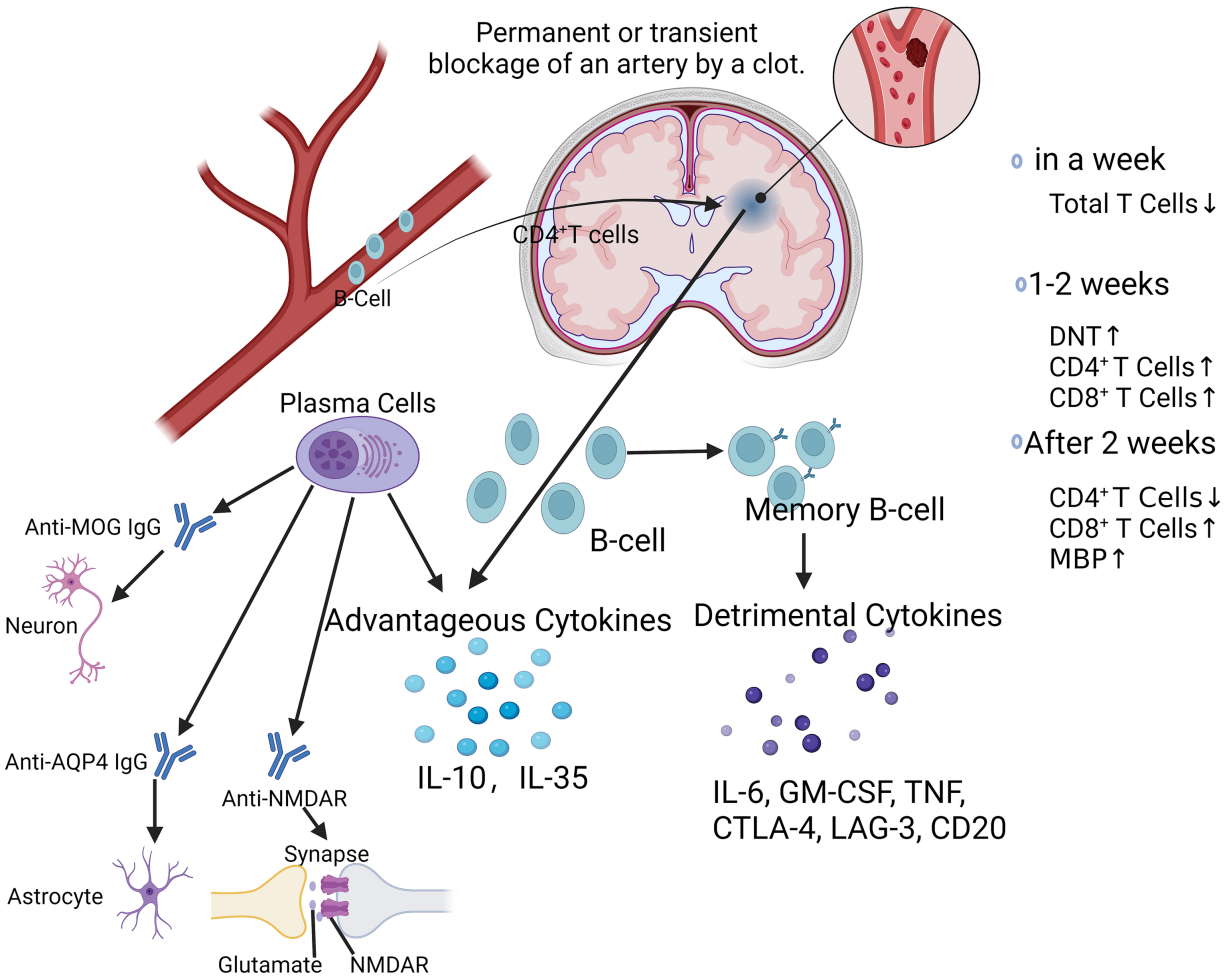
At chronic phase after ischemic stroke. After ischemic stroke, CD4^+^ T-cells drive B-cells to invade the brain and form follicle-like structures. Within 1 week, the total number of T-lymphocytes decreases, followed by a gradual increase in the number of DNT, CD4^+^, and CD8^+^ T-cells over the next 1–2 weeks, and then after 2 weeks, the subpopulations are out of proportion, with a decrease in the number of CD4^+^ T-cells and an increase in the number of CD8^+^ T-cells. Activated B-cells produced favorable cytokines such as IL-10 and IL-35, while memory B-lymphocytes produced unfavorable cytokines such as IL-6, GM-CSF, TNF, CTLA-4, LAG-3, CD20, etc. Plasma cells produce a variety of antibodies such as Anti-MOG IgG, Anti-AQP4 IgG, Anti-NMDAR, which play important regulatory roles in neuronal cells, astrocytes, and synaptic transmission, thereby affecting the progression of a variety of neurological diseases. DNT, double negative T-cells; IL-6, interleukin-6; GM-CSF, granulocyte-macrophage colony-stimulating factor; TNF, tumor necrosis factor; CTLA-4; cytotoxic T-lymphocyte associated protein 4; AQP4, aquaporin 4; MOG-IgG, myelin oligodendrocyte glycoprotein associated immunoglobulin G; NMDAR, N-methyl-D-aspartate receptor.

Multiple cytokines and inflammatory molecules may present harmful effects in the chronic phase of stroke, which need to be further studied and explored (Figure 2). It has also been found that stroke triggers an autoimmune response to CNS antigens, including activation of CD4^+^ and CD8^+^ T-cells as well as CD19^+^ B-cells against neuronal, myelin, and other brain tissue antigens, suggesting that stroke may elicit a complex and dynamic autoimmune response to neuronal antigens, which may exacerbate or ameliorate long-term neuroinflammation (Ortega et al., 2015). Kyra et al. found that myelin basic protein (MBP) antibody titer was the only independent predictor of cognitive decline after stroke by evaluating the Mini-Mental State Examination (MMSE) scores and autoantibody levels in post-stroke patients (Becker et al., 2016). B-lymphocytes may have a pathogenic response to the injured CNS, which may have an impact on the cognitive functioning of patients after a stroke (Doyle et al., 2015; Doyle and Buckwalter, 2017). Luis et al. found that CD4^+^ T-cells are critical for driving B-cells to invade the brain and form follicle-like structures after stroke, which is associated with long-term cognitive impairment after stroke. Reduction of CD4^+^ T-cells by anti-CD4 therapy inhibits brain infiltration by B-cells and may attenuate cognitive impairment after stroke (Weitbrecht et al., 2021). It has also been shown that the absence of B-cells in the splenic marginal zone (MZ) leads to susceptibility to post-stroke infections, which tend to limit the degree of long-term recovery after stroke (McCulloch et al., 2017). Li et al. found that the number of B-cells, regulatory B-cells, and CD8^+^ regulatory T-cells increased significantly after stroke, whereas the number of CD4^+^ regulatory T-cells decreased and then recovered and was highly correlated with infarct volume and neurological scores of the patients (Li et al., 2021). After ischemic cerebral infarction, the total number of T-lymphocytes decreased within 1 week, and the number of double-negative T-cells (DNT), CD4^+^, and CD8^+^ T-cells gradually increased in the brain after 2 weeks, and the proportions of each subpopulation were out of proportion, i.e., the number of CD4^+^ T-cells decreased. After 1–2 weeks, the number of double-negative T-cells (DNT) and CD4^+^ and CD8^+^ T-cells in the brain gradually increased. 2 weeks later, the number of cells gradually recovered, and the proportion of each subpopulation was imbalanced, i.e., the number of CD4^+^ T-cells decreased and the number of CD8^+^ T-cells increased (Liesz and Kleinschnitz, 2016; Kim et al., 2021). It was found that CD8^+^ T-cells appeared as early as 3 h after cerebral infarction, whereas CD4^+^ T-cells appeared as early as 24 h after cerebral infarction, in which CD8^+^ T-cells may be related to neuronal death while CD4^+^ T-cells may play a role in tissue repair (Liesz and Kleinschnitz, 2016). These studies suggest that different subpopulations of lymphocytes are constantly changing in number, type, and function at different times during the stroke course (Figure 2). The positivity of PD-1 and Tim-3 on the surface of CD4^+^ T-cells may be a clinical indicator for determining the prognosis of patients with acute cerebral infarction. Inhibitory checkpoints such as cytotoxic T lymphocyte antigen-4 (CTLA-4), PD-1, and lymphocyte activation gene-3 (LAG-3) are negative modulators that maintain self-tolerance and limit the duration and intensity of immune responses (Liesz and Kleinschnitz, 2016). The PD-1 pathway is central to prevent the activation of autoreactive T-cells (Francisco et al., 2010). We have demonstrated that mice lacking PD-1 have more severe infarcts and worsened inflammatory responses following tMCAO (Ren et al., 2011b), suggesting that the PD-1 pathway is involved in suppressing stroke-induced inflammatory responses. PD-1 is expressed on T-cells (Francisco et al., 2010), monocytes (Zasada et al., 2017), and macrophages (Lu et al., 2019); however, the mechanism by which PD-1 expression alters CECs is still lacking. Positive regulators such as OX40 (CD134) and 401BB function as synergistic stimulators, while other checkpoints such as T-cells are negatively stimulated. Checkpoints such as T-cell immunoglobulin and mucin structural domain-3 (Tim-3) function as stimulatory and inhibitory regulators (Mistry et al., 2017).

B-cells play a dual role in the progression of ischemic stroke, and their deleterious effects are mediated by autologous immunoglobulins, which can be detected in the cerebrospinal fluid of patients with ischemic stroke (Selvaraj et al., 2016; Doyle and Buckwalter, 2017). These specific antibodies, may mediate the chronic inflammatory response in the late stages of ischemic stroke and thus participate in the long-term enhancement of the hippocampus and memory impairment (Doyle et al., 2015). Plasma cells produce a variety of antibodies such as anti-MOG IgG, anti-AQP4 IgG, anti-NMDAR (Figure 2), which play important regulatory roles in neuronal cells, astrocytes, and synaptic transmission, thereby affecting the course of a variety of neurological diseases (Javidi and Magnus, 2019; Lee et al., 2021). Eva et al. found that a more youthful brain appearance was associated with a lower risk of post-stroke neurocognitive deficits (NCD), even in patients who did not show signs of injury within 3 months of stroke. However, B-cell-deficient ischemic stroke mice did not show any improvement in neurological function, whereas B-cell overshoot attenuated the extent of cerebral infarction and improved neurological function, which may be related to the secretion of the anti-inflammatory cytokine IL-10. Eva et al. found that a more youthful brain appearance was associated with a lower risk of post-stroke neurocognitive deficits (NCD), even in patients who did not show signs of injury within 3 months of stroke (Aamodt et al., 2023). Delayed emergence of B-cell and T-cell clusters in the later stages of stroke injury is associated with cognitive decline and may indicate a deleterious adaptive immune response (Tsai et al., 2019). However, B-cell-deficient ischemic stroke mice did not show any improvement in neurological function, whereas B-cell overshoot attenuated the extent of cerebral infarction and improved neurological function, which may be related to the secretion of the anti-inflammatory cytokine IL-10 (Yilmaz et al., 2006; Doyle et al., 2015). Kristian et al. found that genetic defects and pharmacologic elimination of B-cells using anti-CD20 antibodies prevented the appearance of delayed cognitive deficits in a mouse model. In addition, immunostaining of human autopsy tissues showed that B-cell responses occurred in the brains of certain stroke and dementia patients (Doyle et al., 2015). Thus, the role of B-cells in ischemic stroke is contradictory, and the positive effects of IL-10 on the local inflammatory milieu may be counterbalanced by the negative effects of its autocrine antibodies. Bodhankar et al. demonstrated that transplantation of IL-10-secreting B-cells into wild-type mice still produced neuroprotection and also found that it inhibited T-cell infiltration and microglia/monocyte activation in the infarcted area, with a significant increase in Tregs and PD-1 in the peripheral blood and a markedly suppressed peripheral inflammatory milieu (Bodhankar et al., 2014). Sterling et al. found that after stroke, B-cells migrate to remote areas of the brain that are known to produce new neuronal cells and regulate cognitive and motor functions (Ortega et al., 2020). Studies have found that genetic defects and pharmacological ablation of B-lymphocytes with anti-CD20 antibodies can prevent the appearance of late-onset cognitive deficits after ischemic stroke (Doyle et al., 2015). In addition, immunostaining of human postmortem tissues has shown that B lymphocyte responses to stroke also occur in the brains of some stroke and dementia patients (Doyle et al., 2015).

Regulatory B-cells (Breg) are a subpopulation of B-cells with immunosuppressive functions (Mizoguchi et al., 1997). The role of regulatory B-cells in autoimmune diseases has been extensively studied, including experimental autoimmune encephalomyelitis (EAE), rheumatoid arthritis (collagen-induced arthritis (CIA)), type 1 diabetes mellitus (non-obese diabetic mice (NOD mice)), and systemic lupus erythematosus (NZB/W F1 mice), etc. In these animal experiments, Breg plays an important protective role by inhibiting pro-inflammatory T-cell function and promoting Treg proliferation. Breg plays an important protective role by inhibiting pro-inflammatory T-cell function and promoting Treg proliferation, an immunomodulatory process mainly mediated by interleukin-10 (IL-10) (Mizoguchi et al., 1997; Yang et al., 2013; Tedder, 2015). B-cells migrate not only to the site of infarction or stroke but equally to other areas that support motor and cognitive recovery. B-cell-deficient mice have a reduced rate of recovery in these areas after stroke, and B-cells may have a more neurotrophic role (Ortega et al., 2020). Similarly, ischemic stroke is accompanied by a series of immunoinflammatory cascades, and studies by Daien et al. and Flores-Borja et al. have demonstrated a reduction in infarct size and improvement in neurological deficits by attenuating the inflammatory response in a Breg animal model (Flores-Borja et al., 2013; Daien et al., 2014). With the in-depth study of the immunoregulatory function of Treg, the immunoregulatory function of Breg cells has also attracted more and more researchers’ attention. Like Treg, Breg is also a type of endogenous immunomodulatory cell that can inhibit the immune-inflammatory response after cerebral ischemia, thus producing neuroprotective effects (Ren et al., 2011a; Bodhankar et al., 2013).

## 5 The immunotherapy in ischemic stroke

The use of immunomodulators in the treatment of ischemic stroke is an emerging area of research. On the one hand, immunomodulators can affect the activity of microglia, T-cells, and macrophages in ischemic cerebral infarction, thereby regulating immune cell response, reducing harmful inflammation, and promoting beneficial repair processes. On the other hand, immunomodulators also help to protect or restore the integrity of the blood–brain barrier, reduce the permeability of the blood–brain barrier, and prevent the excessive infiltration of peripheral inflammatory cells and inflammatory factors (Ngwa et al., 2022). Although intravenous thrombolysis and endovascular intervention have been proven to be effective methods for the treatment of ischemic cerebral infarction, tPA is the only drug approved by the US Food and Drug Administration (FDA) for the treatment of ischemic stroke, but this drug must be taken within 3 h to be effective. Only a minority of patients are eligible for thrombolytic therapy (Zhao et al., 2017; Ngwa et al., 2022). Given the strict indications and time window, only a small number of patients are eligible for thrombolytic therapy. The limited options for treating core symptoms of stroke, especially acute brain injury after stroke, have led to the need to find a new strategy that can be applied to many clinical patients.

Atorvastatin is an anti-inflammatory drug for the treatment of ischemic cerebral infarction, and the gut microbiota plays an important role in the pathogenesis of ischemic cerebral infarction. Studies have shown that atorvastatin can significantly improve sensory-motor behavior disorders in mice with permanent middle cerebral artery occlusion (pMCAO) and reduce microglia-mediated neuroinflammation by inhibiting the proinflammatory polarization of microglia in the peri-infarct cortex (Zhang et al., 2021). Atorvastatin reversed microbial composition such as increasing the abundance of Lactobacillus and Lactobacillus and decreasing the abundance of mycobacteria, which increased fecal butyric acid levels and promoted intestinal barrier function. At the same time, atorvastatin could regulate intestinal immune function. Such as monocyte chemoattractant protein-1 (MCP-1), tumor necrosis factor-α (TNF-α), and circulating endotoxin (lipopolysaccharide binding protein) decreased, while IL-10 increased (Zhang et al., 2021). It participates in the anti-inflammatory process in cerebral infarction mice by mediating the recovery of intestinal flora, improving intestinal barrier function, and regulating intestinal immune function.

Butylphthalide can be used in the treatment of mild to moderate acute ischemic cerebral infarction and has the effect of anti-apoptosis of nerve cells, but its mechanism of action on inflammation is not clear. Some studies have observed the effect of butylphthalide on the brain tissue of rats with ischemic cerebral infarction, which confirmed its protective effect on ischemic cerebral infarction. The results showed that its mechanism of action was related to the inhibition of nuclear factor-kappa B (NF-κB) κ-light chain to enhance p65 activation. Decreased levels of inflammatory factors IL-6 and TNF-α in brain tissue (Bodhankar et al., 2015).

The immune response is a highly regulated defense system. As a stimulatory or inhibitory signaling pathway triggered by ligand-receptor interactions, immune checkpoints regulate tissue- and pathogen-specific responses and play a key role in cancer, infection, and autoimmune diseases (Mistry et al., 2017). Neuroinflammation in cerebrovascular diseases is associated with neurological deficits. Immuninactivating checkpoint modulators may be a strategy to treat acute and chronic neuroinflammation in cerebrovascular diseases by reducing immune cell aggregation, cytokine secretion, brain edema, and neurodegeneration. Inhibitory checkpoints such as cytotoxic T lymphocyte antigen-4 (CTLA-4), PD-1, and lymphocyte activation gene-3 (LAG-3) are negative regulators that maintain self-tolerance and limit the duration and intensity of immune responses. Positive modulators such as OX40 (CD134) and 401BB have a co-stimulatory function, while other checkpoints such as T-cells are negatively stimulated. Checkpoints such as T-cell immunoglobulin and mucin domain-3 (Tim-3) have stimulatory and inhibitory regulatory functions (Mifsud et al., 2014; Mistry et al., 2017). Therefore, depending on the expression and influence patterns of different immune cell populations and the timing of activity during the inflammatory cascade, each checkpoint may have a unique regulatory signature, and checkpoint-based immunotherapy has great clinical potential (Daien et al., 2014).

Granulocyte colony-stimulating factor (G-CSF), as an endogenous ligand in the central nervous system, has many important functions, such as anti-apoptosis, immune regulation, promoting neuron generation, and angiogenesis. By decreasing pro-apoptotic proteins and increasing anti-apoptotic proteins, G-CSF therapy has a neuroprotective effect on damaged neurons. By decreasing pro-apoptotic proteins and increasing anti-apoptotic proteins, G-CSF therapy has a neuroprotective effect on damaged neurons, reducing acute neuronal degeneration and increasing long-term plasticity after cerebral ischemia (Mifsud et al., 2014; Nikolic et al., 2020). Studies have shown that CB2 receptor agonists can treat ischemic cerebral infarction, and the increased expression of CB2 receptor in CD16^+^ monocytes is related to the severity of cerebral infarction in patients with ischemic cerebral infarction, which can be used as a potential pharmacological target for ischemic cerebral infarction (Greco et al., 2021). Ischemic cerebral infarction disrupts the balance of the brain’s immune system. Inflammation plays an important role in amplifying neuronal damage in the ischemic CNS, and although anti-inflammatory strategies can reduce neuroinflammation, its effect on peripheral immunity should also be considered. Other strategies to modulate peripheral immunity include remote ischemic conditioning, cross-tolerance between organs by altering the composition of immune cells, and post-stroke stem cell therapy (Kim and Cho, 2021).

If combined with thrombolytic therapy, immunomodulation can minimize ischemic injury and immune deficiency, thereby preventing systemic infection and improving survival and the long-term prognosis after ischemic cerebral infarction. However, the occurrence of Immune-Related Adverse Events (irAEs) cannot be ignored (Bai et al., 2021; Verheijden et al., 2023). Common side effects include non-response of the skin, secondary infection of the nervous system, interstitial pneumonia, enteritis, hepatitis, thyroiditis, etc., which can affect almost all organ systems. It limits the benefit of clinical drugs and even endangers the lives of patients in severe cases (Bai et al., 2021; Blum et al., 2023). Skin toxicity is the most common adverse reaction caused by immunotherapy. The main symptoms are rash, blisters, pruritus, etc. (Blum et al., 2023). According to the degree of skin lesions, they can be divided into three grades: mild, moderate, and severe. Most of the adverse reactions are mild. The incidence of immune pneumonia is not high in clinical practice, but the risk is extremely high, and we should pay great attention to it. The main symptoms of immune pneumonia are cough, expectoration, dyspnea, chest pain, etc., and blood oxygen saturation may be less than 90% (Nikolic et al., 2020; Blum et al., 2023). The organs involved in secretory system toxicity include the thyroid, adenohypophysis, adrenal gland, pancreas, and so on. About 10% of patients receiving immunotherapy will experience adverse reactions of the endocrine system, such as hypothyroidism, hyperthyroidism, hypophysitis, thyroiditis, etc., among which hyperthyroidism and hypothyroidism are the most common (Selvaraj et al., 2016). Immune-related hepatotoxicity mainly manifests as elevated alanine aminotransferase (ALT) and/or aspartate aminotransferase (AST), with or without elevated bilirubin. Gastrointestinal adverse reactions are also common, usually manifested as abdominal pain, diarrhea, hematochezia, colitis, and so on (Park et al., 2020; Kim et al., 2021).

In addition, we developed a novel therapeutic approach using blood replacement for the treatment of ischemic stroke in a mouse model. At present, the experimental research on blood replacement for the treatment of ischemic stroke is still in the exploratory stage, and no human experiment has been carried out (Ren et al., 2020). Our previous and current research still focuses on the exploration of the mechanism and implementation strategy of blood exchange therapy to further accurately evaluate the indications and related conditions of this therapy and the prevention of complications. While the results from our study show promising outcomes in animal models, transfusion medicine in a clinical setting is a complex form of medicine that comes with severe outcomes (Ren et al., 2020). These complications are commonly grouped into several different categories, which are pyrogenic, contamination of blood products, hemolytic reactions, ABO incompatibility, and transfusion reactions that are classified as hyper-acute, acute, and chronic (Moore, 1953; Ackfeld et al., 2022). The clinical considerations mentioned above increase the complexity, resources, and time spent on providing treatment for stroke, which is a very time-dependent disease process. The use of blood transfusions for stroke is also complicated by the increase in healthcare utilization combined with trauma activity and unprecedented violence across the country, resulting in a decrease in the number of blood products available (Saillant et al., 2022). Despite these potential drawbacks of using blood transfusions for stroke, there is currently very limited treatment options for stroke that have clinically significant improvements in stroke-related outcomes. Due to the need for more clinically effective treatments for stroke, searching for new avenues for treatment is more warranted.

With the increasing popularity of immunotherapy medications, it is critical to further investigate the processes by which various immunotherapies in other diseases limit the occurrence of major side effects while providing the optimum advantages for patients with ischemic stroke.

## 6 Perspectives

The increased susceptibility to stroke in the elderly is associated with aging of the immune system and immune decline. Prolonged exposure to various antigens leads to clonal depletion of lymphocytes, which affects immunity in the elderly. Studies have focused on T-cell dysfunction, but B-cells are also affected. Changes in B-cells suggest that old patients are in a state characterized by a lack of B-clonal type of immune responses to new foreign pathogens. The data suggest that the loss of naïve B-cells may be a hallmark of immune senescence and may be relevant to human longevity and the evaluation of anti-aging therapies (Colonna-Romano et al., 2008). Studies have shown that older women are more susceptible to stroke, infectious diseases, and dementia compared to other groups, and that aging of the immune system, including changes in B-cells, is associated with the development of a variety of chronic diseases such as cardiovascular disease, autoimmune disorders, and certain types of cancer (Franceschi et al., 2018; Ritzel et al., 2018; Dowery et al., 2021; Jain and Yong, 2022). Aging and age-related diseases have fundamental mechanistic pillars that converge significantly on inflammation. During aging, a chronic, sterile, low-grade inflammation called inflammatory aging develops, leading to the pathogenesis of age-related diseases (Franceschi et al., 2018; Hagen and Derudder, 2020). B-cells play a key role in the immune system, being responsible not only for the production of antibodies but also involved in antigen presentation and immunomodulation. As we age, the number and function of B-cells may change, which may lead to a decreased ability to recognize and respond to pathogens, thereby increasing the risk of disease in the elderly (Franceschi et al., 2018; Ritzel et al., 2018). Relatively few studies have been conducted on B-cells in the aging process; therefore, it is important to further investigate the role of B-cells in the immune aging process and to utilize new biomarkers, including DNA methylation, glycomics, metabolomics, and lipidomics, to assess the biological age of metabolic diseases versus the actual age. Not only is this critical for an in-depth understanding of changes in the immune system of older adults, but it may also provide critical information for the development of new therapeutic strategies to improve the health of older adults (Franceschi et al., 2018).

Min et al. found that the number of early B-cells and their progenitors was reduced in aged mice and that aging negatively affected the development of these cells (Min et al., 2006). This change is specifically in B-cell production and is not due to a general defect in the bone marrow environment or hematopoietic stem cells. With age, mice show a significant decrease in a specific B-cell developmental stage, the pre-B-cell stage. This decrease occurs primarily between 1 and 4 months and 12 and 24 months of age in mice (Stephan et al., 1996; Sadighi Akha, 2018). Research has shown that impaired humoral immune responses and increased autoimmune propensity are closely associated with the development of immune system dysfunction, with the accumulation of a subpopulation of atypical B-cells known as age-associated B-cells (ABCs) being one of the keys to the changes associated with the B-cell compartment. These cells are more common in the elderly and may be associated with increased age-related inflammation. These cells can comprise up to 30% of all mature B-cells by 22 months of age (Hao et al., 2011). They are insensitive to conventional B-cell receptor stimulation but respond to certain immune system receptor stimuli and present antigens efficiently. These findings suggest that with age, while the total number of mature B-cells remains constant, a large proportion of them are occupied by this new type of B-cell (Hao et al., 2011).

It has been shown that with age, B-cell regeneration in the bone marrow decreases, leading to impaired immune function, and this process is associated with the interaction of tumor necrosis factor-α (TNF-α) and insulin-like growth factor-1 (IGF-1) produced by peripheral B-cells, which affects the production of B-cells (Dowery et al., 2021), and this immune-endocrine regulatory circuit that mediates the communication. With age, peripheral B-cells produce increased tumor necrosis factor-alpha (TNF-α), which stimulates the production of insulin-like growth factor-binding protein 1 (IGFBP-1), which binds and sequesters insulin-like growth factor 1 in the circulation (IGF-1) in the circulation, thereby inhibiting its activity in promoting B-cell lymphangiogenesis in the bone marrow, an important hallmark of B-cell senescence (Liesz and Kleinschnitz, 2016; Kim and Cho, 2021). During B-cell depletion in senescent humans and mice, circulating TNF-α is reduced, leading to an increase in IGF-1 and reactivation of B-cell lymphangiogenesis (Ma et al., 2019; Dowery et al., 2021). Studies have shown that IL-10 attenuates neuropathologic changes in stroke and multiple sclerosis and improves prognosis in mice and humans. In stroke, B-cell transfer in normal mice reduced cerebral infarct volume compared to B-cells lacking IL-10 (Ren et al., 2011a; Ortega et al., 2020). This preliminary evidence suggests that certain B-cell subpopulations may aid or prolong neuroinflammation by influencing the behavior of microglia. With age, the balance of immune cells in the peripheral and central nervous system of the body changes, affecting the immune response at sites vulnerable to pathological threats. In addition, the body becomes less responsive to vaccines because of aging, due in part to the attenuation of B-cell function. Therefore, vaccine strategies for the elderly may need to be adapted, e.g., by using boosters or improved vaccine formulations. Overall, the aging process significantly affects B-cell compartments and function in people at high risk for stroke.

## 7 Conclusion

In summary, immune-mediated inflammatory responses play a key regulatory role in the course of ischemic stroke, and a variety of immune cells and inflammatory mediators are involved in ischemic injury or neural repair in ischemic stroke by acting on the lesion and peripheral vasculature and periphery through innate or adaptive immune responses. In recent years, it has been widely recognized that many components of the immune system have multiple roles in ischemic stroke and often shift between beneficial and harmful phenotypes, both of which are influenced by the disease process of ischemic stroke. Both are influenced by the disease process in ischemic stroke and often shift between beneficial or harmful phenotypes. On the one hand, the lymphopenia and immunosuppression that occur after stroke may help to limit the development of autoreactive T-cells, thereby reducing autoimmune attacks on brain tissue. On the other hand, immunosuppression after stroke may increase the risk of infection, which is an important factor influencing prognosis and mortality in neurological diseases. Acute infection negatively affects stroke prognosis by upregulating costimulatory molecules and promoting antigen presentation. This immune response may help protect the brain from further damage. Approximately 30% of stroke survivors develop dementia associated with brain atrophy. Pathologic studies have shown that inflammatory infiltrates can persist for years after a stroke. Monocytes, perivascular leukocyte aggregates, and macrophages have been observed in varying proportions in stroke patients, and T-cells and dendritic cells have been detected, especially in patients with prolonged strokes. This “double-edged” immune phenomenon, coupled with ischemic stroke-induced immunosuppression, calls for the development of effective ischemic stroke therapies based on the intervention of inflammatory damage. However, the development of effective inflammatory immunomodulatory drugs ultimately depends on the investigators’ understanding of how bidirectional communication between the CNS and the immune system occurs, which still requires extensive experimental studies and clinical trials to further develop and validate effective therapeutic strategies.

## Author contributions

SW: Conceptualization, Resources, Writing – original draft, Writing – review & editing. ST: Writing – review & editing. CTP: Writing – review & editing. HH: Writing – review & editing. AMG: Writing – review & editing. HAC: Writing – review & editing. XSR: Conceptualization, Funding acquisition, Resources, Supervision, Writing – original draft, Writing – review & editing.
